# Diagnostic accuracy of signs and symptoms in acute coronary syndrome and acute myocardial infarction: A diagnostic meta-analysis

**DOI:** 10.1080/02813432.2024.2406266

**Published:** 2024-09-22

**Authors:** Willem Raat, Lotte Nees, Bert Vaes

**Affiliations:** Department of Public Health and Primary Care, KU Leuven, Leuven, Belgium

**Keywords:** Acute coronary syndrome, diagnosis, signs, symptoms, meta-analysis, acute myocardial infarction

## Abstract

**Background:**

Acute coronary syndrome (ACS) and acute myocardial infarction (AMI) account for a large portion of cardiovascular deaths. Signs and symptoms for these syndromes, such as chest pain, are non-specific and can be caused by a variety of non-cardiac conditions, especially in low-prevalence settings such as general practice. The diagnostic value of these signs and symptoms can be assessed using diagnostic meta-analyses, but the last one dates from 2012.

**Methods:**

We performed a diagnostic meta-analysis in accordance with PRISMA guidelines. We searched PubMed, Embase and CENTRAL from 2006 to 2024. We included studies that assessed the diagnostic accuracy of thirteen different signs and symptoms. We divided patients into two subgroups (AMI and ACS) on which analysis was performed independently.

**Results:**

We selected 24 articles for inclusion. Our analysis indicates that signs and symptoms have a limited role in the diagnosis of AMI or ACS. The most useful (highest diagnostic odds ratios, DOR) in the diagnosis of AMI were pain radiating to both arms (DOR 2.95 (95%CI 1.57–5.06)), absence of chest wall tenderness (DOR 3.51 (95%CI 1.64–6.61)), pain radiating to the right arm (DOR 5.17 (95%CI 1.77–11.9)) and sweating (DOR 5.75 (95%CI 2.51–11.4)). For ACS these were pain radiating to the right arm (DOR 3.9 (95%CI 0.7–12.6)) and absence of chest wall tenderness (DOR 7.73 (95%CI 2.19–19.8)).

**Conclusion:**

We report the accuracy of thirteen signs and symptoms in the diagnosis of AMI and ACS. These can be useful to calibrate general practitioners’ diagnostic assessment of chest pain in primary care settings.

## Introduction

Acute coronary syndrome (ACS) describes a spectrum of disease originating from interrupted blood supply to the heart muscle, potentially leading to acute myocardial infarction (AMI), where the lack of oxygen leads to loss or damage to heart muscle tissue. Despite substantial progress in many areas of the diagnosis and treatment of acute coronary syndromes, cardiovascular disease remains the leading cause of death globally, with nearly half of these deaths due to ischemic heart disease (1).

ACS and AMI are often accompanied by chest pain, which is a symptom that can also be caused by a variety of non-cardiac conditions. It is therefore crucial for general practitioners (GPs) to be able to differentiate between AMI and other causes of chest pain, especially when patients define chest pain as not very severe or prolonged, but distressing enough to contact their GP (2). The importance of signs and symptoms such as chest pain in the diagnostic assessment of AMI and ACS have been investigated previously: a meta-analysis from 2008 by Bruyninckx et al. of 28 studies with more than 40 000 patients concluded that signs and symptoms alone were not sufficient to diagnose AMI or ACS, but that chest-wall tenderness on palpation could effectively rule out AMI in low-prevalence settings (negative likelihood ratio of 0.23) (2).

Since this meta-analysis is more than fifteen years old and there are new studies as well as meta-analytic methods, we conducted a new meta-analysis to assess the diagnostic value of thirteen signs and symptoms suggestive for AMI or ACS.

## Methods

We performed this diagnostic meta-analysis in accordance with the Preferred Reporting Items for Systematic Reviews and Meta-analyses (PRISMA) 2020 guidelines (3).

We registered this study in the International Prospective Register of Systematic Reviews (PROSPERO) database (ID: CRD42022329976) before data extraction and analysis.

### Data sources and search strategy

We searched PubMed, Embase and CENTRAL from 01 June 2006 to 20 April.2024 to identify applicable studies. We used a search strategy combining MeSH and text terms that encompassed emergency medicine, primary care, and acute coronary syndrome or acute myocardial infarction using Boolean operators (Supplementary Box 1). We also used reference tracking to widen our search.

### Study selection

We included studies that assessed the diagnostic accuracy of the following thirteen signs and symptoms in patients who reported in primary care or emergency department settings with possible ACS or AMI:

- Radiating pain in
  - left arm or shoulder
  - right arm or shoulder
  - both arms
  - neck
  - back
- Epigastric pain
- Oppressive pain (if chest discomfort was not specifically divided in categories of pain versus mere heaviness or tightness, we included these results under oppressive pain.)
- Absence of chest wall tenderness
- Vomiting
- Nausea
- Vomiting and/or nausea
- Sweating
- Sweating- and/or nausea

We excluded studies that corresponded to the following criteria:

- The studied condition was not AMI or ACS
- The study population did not represent the primary care population. For example, they included only patients with elevated cardiac enzymes or ECG changes
- The study was conducted in the coronary care unit (CCU) (selected population)
- The study didn’t include one of the 13 signs or symptoms
- The data was reported in such a way as to impede the construction of a two-by-two cross-table
- Language not in Dutch or English

LN screened all references by title and abstract. Articles were then divided into three groups: inclusion, doubt, or exclusion. The second researcher (BV) screened the inclusion and in doubt group of articles independently and screened a random number of the articles that were excluded by LN. If there was a disagreement, a third reviewer (WR) would be engaged. Full text of the included articles and the articles in doubt were obtained and screened by both researchers (LN and BV) using Rayyan. We kept a list of the excluded articles and reason for exclusion (available upon reasonable request).

### Assessment for study quality and risk of bias

The quality was assessed by one researcher (LN) using the Quality Assessment of Diagnosis Accuracy Studies-2 (QUADAS-2) instrument in Review Manager (RevMan version 5.4, Cochrane training) to evaluate the risk of bias (4).

QUADAS-2 consists of four domains:

- Patient selection: assessment on how patients were selected for the study and the risk of selection bias
- Index test: interpretation of the 13 signs and symptoms
- Reference standard: execution and interpretation of the reference test
- Flow and timing: investigating the sequence in which the index and reference test were performed

Each domain was scored “high”, “unclear” or “low” to determine the risk of bias and applicability (5).

### Data extraction

We extracted the following data from the studies selected for inclusion in the analysis:

- Study design (prospective or retrospective)
- Study setting (primary care, emergency department)
- Patient characteristics (age and sex)
- The index test (one of the thirteen signs and symptoms)
- The sample size and the prevalence of the disease in the research group
- The results of the study as tabulated in absolute numbers in a two-by-two cross-table (or calculated from sensitivity, specificity, or percentages)
- The inclusion and exclusion criteria
- The reference test or standard

### Data analysis

We divided patients in two subgroups (AMI and ACS) on which analysis was performed independently. We used the random effects model of Reitsma et al. (6) to more accurately model the heterogeneity between the different studies (7). We performed analysis using the Mada package in R version 4.3 (8). We used pooled sensitivity (true positive ratio), specificity (true negative ratio or 1- false positive ratio), positive likelihood ratio (posLR), negative likelihood ratio (negLR) and the diagnostic odds ratio (DOR) as outcomes for the clinical utility of different signs and symptoms (9).

The positive likelihood ratio in this manuscript is to be interpreted as the ratio of patients with ACS or AMI (true positives) to patients without ACS or AMI (false positives) in the presence of a symptom (10). Conversely, the negative likelihood ratio is to be interpreted as the ratio of patients with ACS or AMI who do not present with a symptom (false negatives) to patients without ACS or AMI who do not present with a symptom (true negatives). Stated differently, likelihood ratios give the probability that a test result, or in our case a sign or symptom, correctly indicate or exclude disease versus the probability that they are incorrect.

The diagnostic odds ratio can be interpreted as the odds of a positive index test (here the presence of a symptom) in patients with the disease relative to the odds of a positive test in patients without the disease. Mathematically, this can be translated as the positive likelihood ratio divided by the negative likelihood ratio (11).

Unlike sensitivity and specificity, the positive likelihood ratio, negative likelihood ratio and diagnostic odds ratio are not affected by the prevalence of the disease. This contrasts to positive predictive value (PPV) and negative predictive value (NPV), which respectively increase and decrease with higher disease prevalence (12).

In this meta-analysis, we chose not to report positive and negative predictive values since the prevalences of included studies were heterogeneous (13).

## Results

### Study selection

**Figure 1.**
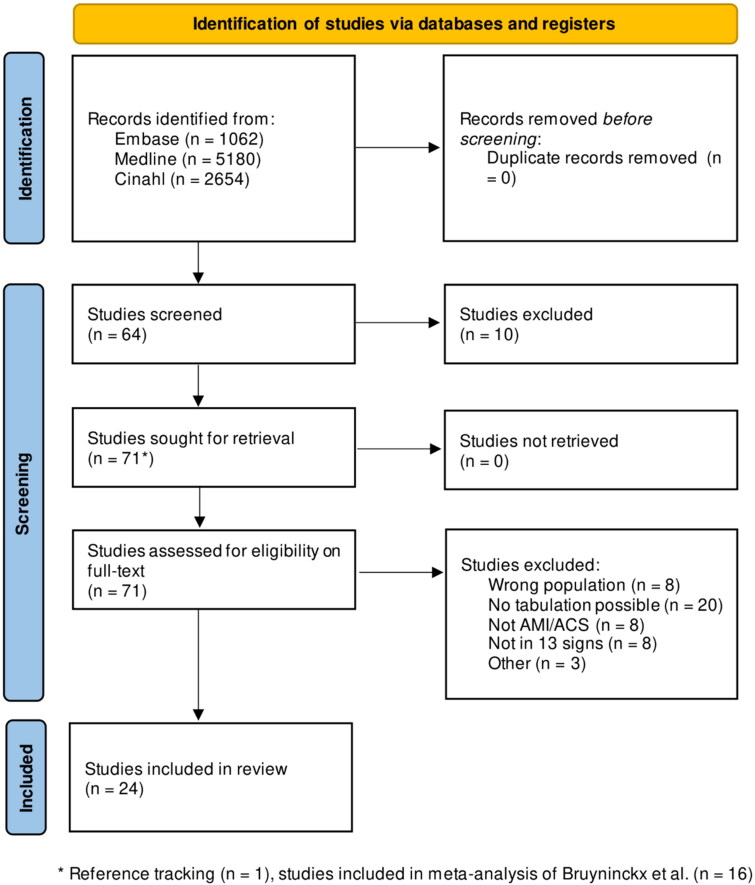
PRISMA Flowchart of selected studies. AMI: acute myocardial infarction; ACS: acute coronary syndrome.

Our initial search yielded 8876 results. 55 articles remained after screening on title and abstract. Reference searching yielded one additional article, and we included 16 articles with appropriate populations (patients not recruited in coronary care units or by cardiologists) from Bruyninckx et al. We selected 24 articles for inclusion in our analyses after full-text review (Figure 1).

**Figure 2.**
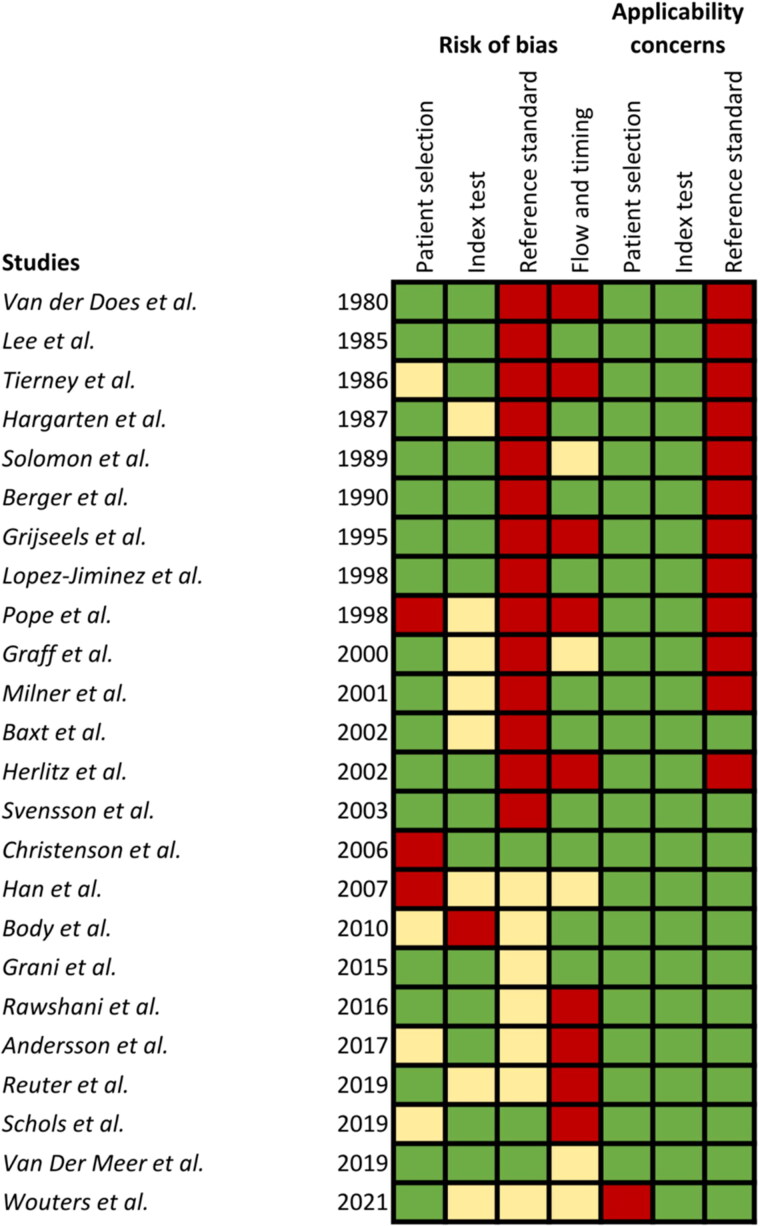
Quality assessment of included studies. Red: high risk of bias; Orange: medium risk of bias; Green: low risk of bias.

Figure 2 presents a quality assessment of the included studies. Overall, the quality of included studies was reasonable. Older studies had less focus on quality assessment or obsolete reference tests.

### Study characteristics

The interventions are summarized in Table 1 and Supplementary Table 1. Overall, there were 72 992 patients from 24 included studies. 11 studies investigated ACS, 10 studies AMI, 3 studies both. The majority of studies (*n* = 14) were conducted in the emergency department. Only two studies were conducted in the general practitioners’ office. Cardiac biomarkers (*n* = 17) and electrocardiogram (*n* = 15) were the most frequently used reference tests to diagnose AMI and ACS.

**Table 1.** Study characteristics and setting.

Study	Year	Design	Sample size	% AMI/ACS	Mean age (years)	Male proportion (%)	Setting
Van der Does (14)	1980	Prospective	1343	7% AMI	54	55%	GP
Lee (15)	1985	Prospective	596	17% AMI 41% ACS	56	48%	ED
Tierney (16)	1986	Prospective	492	12% AMI	NR	NR	ED
Hargarten (17)	1987	Retrospective	401	57% ACS	65	NR	Ambulance service
Solomon (18)	1989	Prospective	7734	14% AMI	NR	50%	ED
Berger (19)	1990	Prospective	278-	36% AMI	NR	69%	ED
Grijseels (20)	1995	Prospective	1005	42% ACS	67	54%	GP
Lopez-Jimenez (21)	1998	Prospective	2694	6% AMI	NR	45%	ED
Pope (22)	1998	Prospective	10689	8% AMI 23% ACS	59	52%	ED
Graff (23)	2000	Prospective	22717	0.85% AMI	NR	NR	ED
Milner (24)	2001	Prospective	531	40% ACS	60	53%	ED
Baxt (25)	2002	Prospective	2204	6% AMI	53	40%	ED
Herlitz (26)	2002	Retrospective	930	14% AMI	71	51%	Ambulance service
Svensson (27)	2003	Prospective	538	29% AMI 57% ACS	69	58%	Ambulance service
Christenson (28)	2006	Prospective	769	21% ACS	58	62%	ED
Han (29)	2007	Retrospective	10126	8% ACS	NR	44.4%	ED
Body (30)	2010	Prospective	796	19% AMI	59	60.4%	ED
Gräni (31)	2015	Prospective	121	12% ACS	NR	60.3%	ED
Rawshani (32)	2016	Retrospective	2285	12% ACS	67	51%	Emergency call centre
Andersson (33)	2017	Retrospective	1702	26 % AMI	NR	55.9%	Ambulance service
Reuter (34)	2019	Prospective	2485	16 % ACS	58	56.3 %	Emergency call centre
Schols (35)	2019	Prospective	243	18% ACS	64	52.3%	ED
van der Meer (36)	2019	Retrospective	518	9% ACS	62	46.7%	Out-of-hours primary care calls
Wouters (37)	2021	Retrospective	1795	11% ACS	59	44.7%	Out-of-hours primary care calls

AMI: acute myocardial infarction; ACS: acute coronary syndrome; GP: general practitioner ED: emergency department; NR: not reported.

### Diagnostic accuracy of signs and symptoms

**Figure 3.**
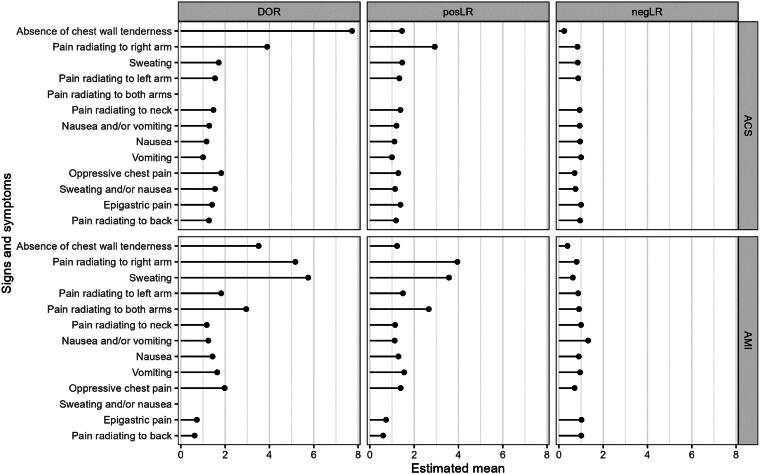
Cleveland plot of estimated means for diagnostic odds ratio (DOR), positive likelihood ratio (posLR), and negative likelihood ratio (negLR). ACS: acute coronary syndrome; AMI: acute myocardial infarction.

**Figure 4.**
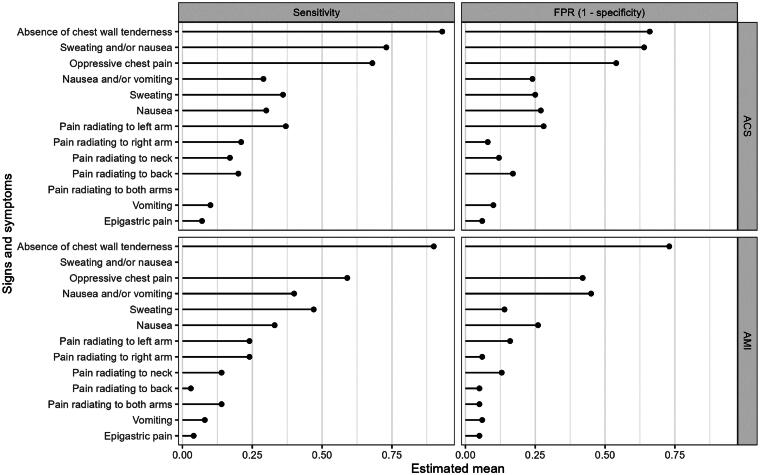
Cleveland plot of estimated means for sensitivity (also known as true positive rate) and false positive rate (FPR). The false positive rate is equal to 1 - specificity.

Table 2, Figures 3 and  4 provide a summary and visualization of the clinical utility of the thirteen signs and symptoms. For AMI the three signs and symptoms with the highest positive likelihood ratio were pain radiating to the right arm (posLR 3.95 (95%CI 1.67–7.53)), sweating (posLR 3.95 (95%CI 1.67–7.53)) and pain radiating to both arms (posLR 2.66 (95%CI 1.52–4.31)). For ACS positive likelihood ratios were lower and less pronounced generally, although the ranking was similar, with pain radiating to the right arm (posLR 2.93 (95%CI 0.72–7.38)), sweating (posLR 1.46 (95%CI 1.28–1.67)) and absence of chest wall tenderness (posLR 1.45 (95%CI 1.1–2.12)) scoring highest.

**Table 2.** Summary table of mean estimates.

Sign	Syndrome	Studies	DOR	posLR	negLR	Sensitivity	False positive rate
Pain radiating to back	ACS	4	1.27 (0.62–2.33)	1.18 (0.66–1.85)	0.96 (0.77–1.07)	0.2 (0.07–0.46)	0.17 (0.08–0.34)
	AMI	2	0.62 (0.15–1.7)	0.6 (0.15–1.4)	1.01 (0.83–1.06)	0.03 (0–0.43)	0.05 (0.01–0.33)
Epigastric pain	ACS	3	1.41 (0.38–3.73)	1.38 (0.43–3.54)	1 (0.95–1.13)	0.07 (0.06–0.09)	0.06 (0.02–0.19)
	AMI	5	0.72 (0.46–1.07)	0.73 (0.48–1.07)	1.02 (1–1.05)	0.04 (0.01–0.1)	0.05 (0.02–0.12)
Sweating and/or nausea	ACS	1	1.54 (1.15–2.03)	1.14 (1.05–1.25)	0.75 (0.61–0.91)	0.73 (0.68–0.77)	0.64 (0.59–0.68)
Oppressive chest pain	ACS	7	1.82 (1.22–2.62)	1.28 (1.06–1.61)	0.71 (0.6–0.87)	0.68 (0.58–0.76)	0.54 (0.37–0.7)
	AMI	7	1.98 (1.63–2.39)	1.39 (1.3–1.49)	0.71 (0.61–0.8)	0.59 (0.5–0.68)	0.42 (0.36–0.49)
Vomiting	ACS	1	1 (0.86–1.16)	1 (0.88–1.14)	1 (0.98–1.01)	0.1 (0.09–0.11)	0.1 (0.09–0.11)
	AMI	4	1.64 (0.76–-3.08)	1.55 (0.77–2.64)	0.96 (0.85–1.01)	0.08 (0.03–0.23)	0.06 (0.03–0.1)
Nausea	ACS	1	1.16 (1.05–1.28)	1.11 (1.04–1.19)	0.96 (0.93–0.99)	0.3 (0.28–0.32)	0.27 (0.26–0.28)
	AMI	3	1.43 (1.2–1.69)	1.29 (1.12–1.5)	0.9 (0.86–0.94)	0.33 (0.24–0.44)	0.26 (0.17–0.38)
Nausea and/or vomiting	ACS	7	1.28 (1–1.61)	1.2 (1–1.46)	0.94 (0.9–1)	0.29 (0.21–0.38)	0.24 (0.15–0.36)
	AMI	3	1.24 (0.14–4.89)	1.12 (0.43–3.21)	1.32 (0.63–3.42)	0.4 (0.25–0.57)	0.45 (0.12–0.83)
Pain radiating to neck	ACS	4	1.47 (1.06–1.99)	1.38 (1.05–1.75)	0.94 (0.85–0.99)	0.17 (0.08–0.33)	0.12 (0.06–0.23)
	AMI	2	1.17 (0.51–2.31)	1.14 (0.57–2.1)	1 (0.91–1.12)	0.14 (0.1–0.19)	0.13 (0.07–0.23)
Pain radiating to both arms	AMI	1	2.95 (1.57–5.06)	2.66 (1.52–4.31)	0.91 (0.84–0.97)	0.14 (0.09–0.2)	0.05 (0.04–0.07)
Pain radiating to left arm	ACS	4	1.54 (1.31–1.79)	1.33 (1.22–1.44)	0.87 (0.78–0.94)	0.37 (0.24–0.52)	0.28 (0.19–0.39)
	AMI	7	1.82 (0.69–3.94)	1.49 (0.72–2.54)	0.87 (0.52–1.04)	0.24 (0.04–0.71)	0.16 (0.04–0.49)
Sweating	ACS	7	1.71 (1.46–2)	1.46 (1.28–1.67)	0.85 (0.79–0.9)	0.36 (0.26–0.48)	0.25 (0.17–0.35)
	AMI	7	5.75 (2.51–11.4)	3.57 (1.75–6.9)	0.63 (0.57–0.71)	0.47 (0.4–0.53)	0.14 (0.06–0.29)
Pain radiating to right arm	ACS	1	3.9 (0.7–12.6)	2.93 (0.72–7.38)	0.84 (0.55–1.04)	0.21 (0.07–0.49)	0.08 (0.04–0.15)
	AMI	3	5.17 (1.77–11.9)	3.95 (1.67–7.53)	0.8 (0.62–0.95)	0.24 (0.12–0.42)	0.06 (0.05–0.09)
Absence of chest wall tenderness	ACS	3	7.73 (2.19–19.8)	1.45 (1.1–2.12)	0.24 (0.1–0.51)	0.93 (0.89–0.95)	0.66 (0.44–0.83)
	AMI	5	3.51 (1.64–6.61)	1.23 (1.11–1.37)	0.39 (0.2–0.68)	0.9 (0.8–0.95)	0.73 (0.64–0.79)

DOR : diagnostic odds ratio; posLR: positive likelihood ratio; negLR: negative likelihood ratio. 95% confidence intervals are indicated between brackets.

In terms of negative likelihood ratios, the three lowest (and therefore accurate) ratios for AMI were absence of chest wall tenderness (negLR 0.39 (95%CI 0.2–0.68)), sweating (negLR 0.63 (95%CI 0.57–0.71)) and oppressive chest pain (negLR 0.71 (95%CI 0.61–0.8)). For ACS, negative likelihood ratios were generally higher and thus less accurate (as was the case for positive likelihood ratios), except for the absence of chest wall tenderness, where the negative likelihood ratio was even lower (negLR 0.24 (95%CI 0.1–0.51)). Oppressive chest pain (negLR 0.71 (95%CI 0.6–0.87)) and sweating and/or nausea (negLR 0.75 (0.61–0.91)) completed the top three.

The likelihood ratios are reflected in the rather low diagnostic odds ratios, especially for ACS. Only pain radiating to the right arm (DOR 3.9 (95%CI 0.7–12.6)) and absence of chest wall tenderness (DOR 7.73 (95%CI 2.19–19.8)) have a ratio higher than 2 for ACS. For AMI, these are pain radiating to both arms (DOR 2.95 (95%CI 1.57–5.06)), absence of chest wall tenderness (DOR 3.51 (95%CI 1.64–6.61)), pain radiating to right arm (DOR 5.17 (95%CI 1.77-11.9)) and sweating (DOR 5.75 (95%CI 2.51–11.4)).

## Discussion

The results of our diagnostic meta-analysis indicate that signs and symptoms have a limited role in the diagnosis of acute myocardial infarction (AMI) or acute coronary syndrome (ACS). The most useful signs and symptoms (highest diagnostic odds ratios) in the diagnosis of AMI were pain radiating to both arms (DOR 2.95 (95%CI 1.57–5.06)), absence of chest wall tenderness (DOR 3.51 (95%CI 1.64–6.61)), pain radiating to the right arm (DOR 5.17 (95%CI 1.77–11.9)) and sweating (DOR 5.75 (95%CI 2.51–11.4)). For ACS these were pain radiating to the right arm (DOR 3.9 (95%CI 0.7–12.6)) and absence of chest wall tenderness (DOR 7.73 (95%CI 2.19–19.8)).

Our study differs from two previous meta-analyses on this topic. First, with regards to methods. In contrast to Haasenritter et al. (38) and Bruyninckx et al. (2) we did not include patients admitted in a cardiac care unit in our analysis, since we wanted to focus on low prevalence settings. Haasenritter et al. also included stable coronary disease and major cardiac events as target diseases. In contrast to Bruyninckx et al. we used a bivariate model to calculate odds ratios, which is the most commonly used method for diagnostic meta-analysis since 2005 (39). In addition, we included ten additional studies in our analysis that were not included in these previous meta-analyses. Second, with regard to results. In the case of AMI, we could calculate a DOR for pain radiating to both arms due to the inclusion of new studies, in contrast to Bruyninckx et al. This symptom was not included in the analysis of Haasenritter et al. whereas our findings indicate it is among the most useful. With regards to the other three signs and symptoms for AMI we found a lower DOR for chest wall tenderness than that reported in Bruyninckx et al. (DOR 6.39), and higher DORs for pain radiating to the right arm (Bruyninckx DOR 3.22, Haasenritter DOR 5.1) and sweating (Bruyninckx DOR 4.54). In the case of ACS, we calculated lower values for the presence of pain radiating to the right arm and the absence of chest wall tenderness than Bruyninck et al. (DOR 4.40 and 8.29 respectively). It should be noted that the differences between our results and those reported by Bruyninckx et al. and Haasenritter et al. are minor, and correspond to the same order of magnitude, despite the differences in study selection and the inclusion of newer studies.

We believe our findings contribute to the evaluation of chest pain in primary care when used in conjunction with a scoring system stratifying risk in this setting, such as the HEART score (40), which also depend on an ECG and troponin measurement. These last two diagnostic tools are not always readily available to general practitioners, particularly during house calls for vulnerable patient populations who can present with atypical signs and symptoms (such as elderly women). A glance at the visualization of the strength of different signs and symptoms can help calibrate general practitioners’ assessment at the point-of-care.

### Strengths and limitations

The main strength of this study is the update and reinforcement of previous research with new studies. Our study has several limitations inherent to diagnostic meta-analysis. First, there was a degree of heterogeneity in study populations and reference testing. We tried to minimize this by only selecting patients in primary care or emergency department settings (and excluding patients recruited by cardiologists or coronary care unit settings) and limiting our disease outcome to acute myocardial infarction and acute coronary syndrome. Although patients presenting with symptoms suggestive of ACS or AMI in primary might differ in characteristics from those presenting in emergency care settings, we felt that pooling was justified because of the disparity in strength of primary care health systems in the Western settings, where there are sharp differences in the number of practicing primary care physicians, after-hours availability of primary care and gatekeeping practices (41–44). Second, we may have underestimated the prevalence of several symptoms, since some studies reported missing data for a small number of patients with regards to the occurrence of signs and symptoms. Third, several inferences were drawn from a small study sample size, sometimes from a single study. For example, there was only one study available on the accuracy of pain radiating to the right arm in ACS.

## Conclusion

We report the accuracy of thirteen signs and symptoms in the diagnosis of acute myocardial infarction and acute coronary syndrome. These can be useful to calibrate general practitioners’ diagnostic assessment of chest pain in primary care settings.

## Supplementary Material

Supplementary_data.docx

## Data Availability

The data underlying this study are available in the published article and its online supplementary material.
