# Supplementary material for: Diagnostic accuracy of signs and symptoms in acute coronary syndrome and acute myocardial infarction: A diagnostic meta-analysis
Source: Scand J Prim Health Care. 2024 Sep 22;43(1):111–9. doi: 10.1080/02813432.2024.2406266 (PMC11834805; doi:10.1080/02813432.2024.2406266)
Supplement: Supplementary_data.docx [file IPRI_A_2406266_SM2275.docx]

Supplementary data

| - MEDLINE: (“Physicians, Family”[MeSH] OR “Emergency Service, Hospital”[MeSH] OR “Emergency Medical Services”[MeSH] OR “Emergency Medicine”[MeSH]) AND (“Chest Pain”[MeSH] OR “Myocardial Ischemia”[MeSH]).  - CINAHL: ("emergency medicine" OR "emergency service" OR "emergency physicians" OR "emergencies or emergency" OR "emergency care" OR "emergency medical services" OR "family physician" OR "prehospital care" AND "angina pectoris" OR "chest pain" OR "myocardial infarction" OR "myocardial ischemia") In this search we excluded Medline records.  - EMBASE:(‘general practitioner’ OR ‘emergency health service’ OR ‘emergency ward’ OR ‘emergency medicine’) AND (‘thorax pain’ OR ‘heart muscle ischemia’) This search was limited to terms indexed in the article as ‘major focus’. |
| --- |

Box S1 Literature search strategy

Table S1 Study inclusion criteria, index test and reference test. AMI = acute myocardial infarction, ACS = acute coronary syndrome, GP = general practitioner, ED = emergency department, ECG = electrocardiogram, yr = year, h = hour, BP = blood pressure, PTCA = percutaneous transluminal coronary angioplasty, pt = patient, a-HBDH =a-hydroxybutyrate dehydrogenase, GOT = glutamic oxaloacetic transaminase, GPT = glutamic pyruvic transaminase, CK = creatine kinase, LDH = lactate dehydrogenase, WHO = world health organization, trop = troponin, PCI = percutaneous coronary intervention, MRI = magnetic resonance imaging.

| **Study** | **Inclusion criteria** | **Index test** | **Reference test** |
| --- | --- | --- | --- |
| Van der Does (14) | Recent chest pain or dyspnea, palpitations or dizziness or syncope, upper abdominal pain or mood changes | Sweating | ECG, cardiac biomarkers (a-HBDH, GOT, CK), symptoms |
| Lee (15) | Chief complaint of anterior, precordial or left lateral chest pain | Epigastric pain, oppressive pain, absence of chest wall tenderness | ECG, cardiac biomarkers (LDH, CK), scintiscan |
| Tierney (16) | Anterior chest pain as one of their complaints | Pain in right arm, oppressive pain, nausea and/or vomiting, sweating, absence of chest wall tenderness | ECG, cardiac biomarkers (CK, LDH) |
| Hargarten (17) | ‘Stable’ chest pain | Vomiting and/or nausea, sweating | ECG, cardiac biomarkers (CK, LDH), autopsy, scintiscan |
| Solomon (18) | Chief complaint of anterior, precordial or left lateral chest pain | Oppressive pain, sweating, absence of chest wall tenderness | ECG, cardiac biomarkers (CK, LDH), scintiscan, sudden unexplained death within 72 hr |
| Berger (19) | Admitted to the hospital, complaining chiefly of chest pain | Pain in left arm, pain in right arm, oppressive pain, vomiting and/or nausea | ECG, cardiac biomarkers (CK), symptoms |
| Grijseels (20) | Symptoms of possible cardiac origin seen by GP and transferred | Sweating and/or nausea | ECG, cardiac biomarkers, history, stress test, coronary angiography |
| Lopez-Jimenez (21) | Chief complaint of chest pain | Oppressive pain, absence of chest wall tenderness | ECG, cardiac biomarkers (CK, LDH), scintiscan, sudden unexplained death within 72 hr |
| Pope (22) | Chief complaint chest, left arm, jaw or epigastric pain or discomfort, dyspnea, dizziness, palpitations or other symptoms suggestive of acute ischemia | Epigastric pain, vomiting, nausea | WHO criteria |
| Graff (23) | All patients with possible AMI where a rapid ECG was performed | Epigastric pain, pain in back, vomiting | WHO criteria |
| Milner (24) | >45 yr and one symptom suggestive of ACS, or 18–44 yr if diabetes and two risk factors | Pain in left arm, pain in back, nausea and/or vomiting, sweating | ECG, cardiac biomarkers (CK) |
| Baxt (25) | Anterior chest pain prompting an ECG | Pain in left arm, pain in neck, oppressive pain, nausea and/or vomiting, sweating | European Society of Cardiology criteria |
| Herlitz (26) | Chest pain or slightest suspicion of an acute coronary syndrome | Epigastric pain, nausea, vomiting, sweating | ECG, cardiac biomarkers (CK), symptoms |
| Svensson (27) | Chest pain or discomfort >15 min, within last 6 h, dyspnea, or any condition suggesting acute coronary syndrome | Sweating | ECG, cardiac biomarkers (CK, troponin), symptoms |
| Christenson (28) | Primary complaint of anterior or lateral chest pain | Pain in left arm, pain in neck | ECG, cardiac biomarkers (CK, troponin), symptoms, stress test, PCI, coronary angiography, death with no other definite cause |
| Han (29) | > 18 yr and suspected of ACS, also chest pain, patients with dyspnea, light-headedness, dizziness, and weakness | Oppressive pain, pain in left arm | Cardiac biomarkers (CK, troponin), death within 30 days, PCI |
| Body (30) | Suspected cardiac chest pain, within the last 24 h | Radiating pain in left arm/shoulder, right arm/shoulder, both arms, neck and back, epigastric pain , oppressive pain, vomiting, nausea, sweating, absence of chest wall tenderness | Cardiac biomarkers (troponin) |
| Gräni (31) | Self-reported acute chest pain | Absence of chest wall tenderness, epigastric pain, oppressive pain, radiation to left arm, right arm, back, neck, nausea, sweating | ECG, cardiac biomarkers (troponin) |
| Rawshani (32) | Symptoms of pain or discomfort in the chest | Nausea/vomiting, sweating | Diagnosis from medical file |
| Andersson (33) | Pain or discomfort that aroused suspicion of ACS and reported pain ≥4 on a visual analogue scale (VAS) | Nausea/vomiting | Diagnosis from medical file |
| Reuter (34) | Non-traumatic chest pain | Oppressive pain | ECG, cardiac biomarkers (troponin), symptoms, stress test, stress echocardiography, cardiac MRI, coronary angiography |
| Schols (35) | Patients referred for suspected ACS | Oppressive pain, absence of chest wall tenderness | Diagnosis from medical file |
| van der Meer (36) | Chest discomfort | Oppressive pain, pain in back, pain in neck, nausea/vomiting, sweating | Diagnosis from medical file |
| Wouters (37) | Chest discomfort (chest pain, pressure, tightness or discomfort) | Oppressive pain, pain in back, pain in neck, sweating, nausea/vomiting | ECG, cardiac biomarkers (troponin), symptoms |
